# *Alcaligenes* lipid A as a sublingual adjuvant to augment protective immune responses in the respiratory and gastrointestinal tracts

**DOI:** 10.1093/intimm/dxaf066

**Published:** 2025-11-26

**Authors:** Ken Yoshii, Zilai Liu, Atsushi Shimoyama, Yuki Hirayama, Keigo Iemitsu, Eri Node, Koji Hosomi, Hiroshi Kiyono, Koichi Fukase, Jun Kunisawa

**Affiliations:** Laboratory of Vaccine Materials and Laboratory of Gut Environmental System, Microbial Research Center for Health and Medicine, National Institutes of Biomedical Innovation, Health, and Nutrition (NIBN), Osaka 567-0085, Japan; Laboratory of Vaccine Materials and Laboratory of Gut Environmental System, Microbial Research Center for Health and Medicine, National Institutes of Biomedical Innovation, Health, and Nutrition (NIBN), Osaka 567-0085, Japan; Graduate School of Pharmaceutical Sciences, The University of Osaka, Osaka 565-0871, Japan; Graduate School of Science, The University of Osaka, Osaka 560-0043, Japan; Laboratory of Vaccine Materials and Laboratory of Gut Environmental System, Microbial Research Center for Health and Medicine, National Institutes of Biomedical Innovation, Health, and Nutrition (NIBN), Osaka 567-0085, Japan; Graduate School of Pharmaceutical Sciences, The University of Osaka, Osaka 565-0871, Japan; Laboratory of Vaccine Materials and Laboratory of Gut Environmental System, Microbial Research Center for Health and Medicine, National Institutes of Biomedical Innovation, Health, and Nutrition (NIBN), Osaka 567-0085, Japan; Graduate School of Medicine, The University of Osaka, Osaka 565-0871, Japan; Laboratory of Vaccine Materials and Laboratory of Gut Environmental System, Microbial Research Center for Health and Medicine, National Institutes of Biomedical Innovation, Health, and Nutrition (NIBN), Osaka 567-0085, Japan; Laboratory of Vaccine Materials and Laboratory of Gut Environmental System, Microbial Research Center for Health and Medicine, National Institutes of Biomedical Innovation, Health, and Nutrition (NIBN), Osaka 567-0085, Japan; Graduate School of Veterinary Science, Osaka Metropolitan University, Osaka 598-8531, Japan; Division of Gastroenterology, Department of Medicine, University of California San Diego (UCSD) School of Medicine, UC San Diego, San Diego, CA 92093, USA; Chiba University (CU)—UCSD Center for Mucosal Immunology, Allergy and Vaccines (cMAV), UC San Diego School of Medicine, UC San Diego, San Diego, CA 92093, USA; Future Medicine Education and Research Organization, Chiba University, Chiba 260-8670, Japan; Department of Human Mucosal Vaccinology, Chiba University Hospital, Chiba 260-8670, Japan; Synergy Institute for Futuristic Mucosal Vaccine Research and Development (cSIMVa), Chiba University, Chiba 260-8670, Japan; Graduate School of Science, The University of Osaka, Osaka 560-0043, Japan; Laboratory of Vaccine Materials and Laboratory of Gut Environmental System, Microbial Research Center for Health and Medicine, National Institutes of Biomedical Innovation, Health, and Nutrition (NIBN), Osaka 567-0085, Japan; Graduate School of Pharmaceutical Sciences, The University of Osaka, Osaka 565-0871, Japan; Graduate School of Science, The University of Osaka, Osaka 560-0043, Japan; Graduate School of Medicine, The University of Osaka, Osaka 565-0871, Japan; International Research and Development Center for Mucosal Vaccines, The Institute of Medical Science, The University of Tokyo, Tokyo 108-8639, Japan; Graduate School of Medicine, Kobe University, Kobe 650-0017, Japan; Research Organization for Nano and Life Innovation, Waseda University, Tokyo 162-0041, Japan; Graduate School of Dentistry, The University of Osaka, Osaka 565-0871, Japan

**Keywords:** IgA antibody, sublingual immunization, Th17 response

## Abstract

We previously identified *Alcaligenes* as symbiotic bacteria residing within Peyer’s patches and demonstrated that their primary components, lipopolysaccharides, and their active center, lipid A, are excellent adjuvants for mucosal vaccination. Here, we evaluated the effectiveness of *Alcaligenes*-derived lipid A as an adjuvant for sublingual immunization, a novel vaccination route. Mice sublingually immunized with *Alcaligenes* lipid A and ovalbumin (OVA) showed enhanced production of OVA-specific IgA in both the respiratory and gastrointestinal tracts. In addition, increased serum levels of OVA-specific and IgG antibodies were elicited through germinal center reactions in the draining lymph nodes without excessive inflammation at the administration sites. These results demonstrated superior efficacy not previously achieved through other routes of administration (e.g. intranasal, subcutaneous, intramuscular administration) or by existing adjuvants (e.g. CpG-ODN). In addition, sublingual immunization with cholera toxin B subunit (CTB) and lipid A led to an elevated CTB-specific IgG response in the systemic compartment and an elevated IgA response in the intestinal tract, effectively suppressing the diarrhea induced by oral challenge with cholera toxin. Furthermore, immunization with pneumococcal surface protein A (PspA) plus *Alcaligenes* lipid A elicited strong PspA-specific CD4^+^ T cell proliferation and Th17 responses, as well as IgA and IgG responses, in both the respiratory tract and the systemic compartment. These effects enhanced pneumococcal clearance in the lungs and subsequent protection against *Streptococcus pneumoniae* infection. Together, our findings suggest that *Alcaligenes*-derived lipid A is a potent sublingual vaccine adjuvant with potential efficacy against both respiratory and intestinal infectious diseases.

## Teaser

Sublingual lipid A is a potent mucosal adjuvant

## Graphical Abstract

**Graphical Abstract:**
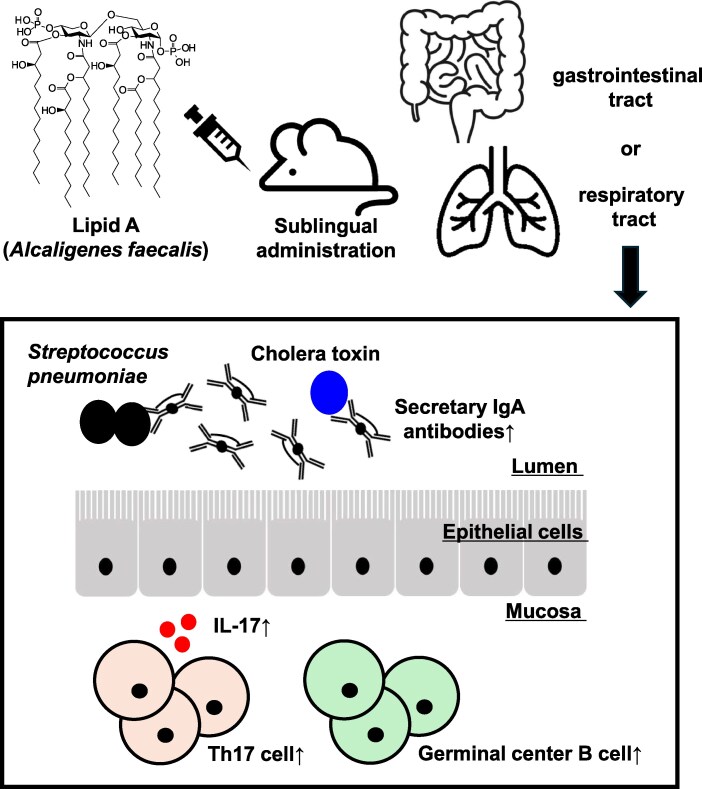

## Introduction

Through their biological defenses, such as the epithelium, mucus, and antimicrobial peptides, mucosal tissues serve as critical barriers against colonization and invasion by various microbes (1). Several immune-related cell types, including M cells (specialized for antigen uptake and transport into gut-associated lymphoid tissue), dendritic cells (DCs), T cells, and B cells, contribute to the formation of highly specialized mucosal immune systems (1, 2). Among these, antigen-specific secretory IgA (SIgA) antibodies, present on the surface of mucosal tissues, play a predominant role in humoral immune responses, protecting the host from pathogens and their derived toxins (1–3). Mucosal vaccines have the unique ability to induce antigen-specific IgA antibody production, making them particularly effective for preventing mucosa-related infectious diseases compared with injectable vaccines, which primarily elicit systemic immune responses, such as IgG antibody production in the bloodstream.

Sublingual administration is an advantageous method of drug delivery. Drugs administered via this route can rapidly and efficiently enter the systemic circulation through the dense network of blood vessels beneath the thin sublingual epithelium, bypassing the enterohepatic first-pass effect. This results in faster and more efficient absorption than with traditional oral delivery systems (4). In addition, a network of lymphatic vessels under the sublingual epithelium facilitates immune-response induction in the sublingual mucosa. Resident immune cells, such as DCs in the sublingual mucosa, can capture antigens and migrate to regional lymph nodes, subsequently spreading to distant lymph nodes, thereby inducing humoral responses at other mucosal sites (e.g. in the respiratory tract and intestinal tract) and the systemic compartment (5, 6). Considering the limitations of currently licensed mucosal vaccines, such as oral vaccines that primarily induce strong immune responses in the intestinal tract, sublingual vaccines hold promise as more versatile and effective types of mucosal vaccines.

Adjuvants play a crucial role in enhancing the immune responses induced by vaccines, thus addressing the limited immunogenicity of vaccine antigens (7). This is particularly important for mucosal vaccines to ensure the induction of protective immunity, rather than immune tolerance, which prevents excessive and potentially harmful immune responses and might therefore prevent the induction of strong immunity (8). Various studies have demonstrated that certain microbial components and metabolites can influence host immunity. One key mechanism involves the interaction between microbial ligands and pattern recognition receptors, such as toll-like receptors (TLRs), which amplify immune responses (9, 10). For instance, lipopolysaccharide (LPS), a major component of the outer membrane of most gram-negative bacteria, is a well-characterized TLR4 agonist that stimulates DCs and promotes the secretion of immune-related cytokines (11). However, only a few adjuvants have been examined clinically for mucosal vaccines, underscoring the need to develop new, effective, and safe adjuvants.

In our previous study, we identified *Alcaligenes*, commensal bacteria that specifically reside in Peyer’s patches, a component of mucosa-associated lymphoid tissues (12, 13). Our study demonstrated that *Alcaligenes*-derived LPS acts as a weak TLR4 agonist, inducing lower levels of nitric oxide and IL-6 production in bone-marrow-derived DCs than those induced by *Escherichia coli*-derived LPS. Unlike *E. coli* LPS, *Alcaligenes* LPS does not induce excessive inflammation (14–16). The unique structure of its core component has been shown in lipid A (17). Chemically synthesized *Alcaligenes* lipid A activates DCs to secrete cytokines such as IL-6 and IL-2 (16, 18) for enhancing antigen-specific immune responses—particularly Th17 responses (16, 18, 19). Furthermore, when *Alcaligenes* lipid A was used as a nasal vaccine adjuvant, it induced antigen-specific SIgA responses, suggesting its potential as an effective vaccine adjuvant. However, whether *Alcaligenes* lipid A could enhance immune responses through sublingual administration remained unclear.

Here, we further explored the use of *Alcaligenes* lipid A as a sublingual vaccine adjuvant. Our findings demonstrate that *Alcaligenes* lipid A effectively induces antigen-specific IgA responses in both the respiratory [pneumococcal surface protein A (PspA)-specific] and intestinal [cholera toxin (CT)-specific] tracts, providing protection against *Streptococcus pneumoniae* infection and CT-induced diarrhea.

## Methods

### Mice

Female BALB/c mice (age, 8 weeks, CLEA Japan, Tokyo, Japan) were purchased and kept for 1 week before experiments were initiated. All animal experiments were conducted in accordance with the Animal Care and Use Committee guidelines of the National Institutes of Biomedical Innovation, Health, and Nutrition (NIBN) and the Committee on the Ethics of Animal Experiments of NIBN (approval nos. DSR04-37R7 and DSR04-38R7).

### Preparation of *Alcaligenes* lipid A

*Alcaligenes* lipid A which was chemically synthesized as previously described (17) or purchased (PEPTIDE Institute Inc., Osaka, Japan) was dissolved in dimethyl sulfoxide (Nacalai Tesque, Kyoto, Japan), and stored at −30°C.

### Preparation of PspA protein and endotoxin removal

The PspA gene was amplified by polymerase chain reaction (PCR) and cloned into pET16b plasmid (Novagen, Darmstadt, Germany), as previously described, to yield pET16b-PspA plasmid (20). To obtain PspA recombinant proteins, the plasmids were transformed into *E. coli* strain BL21 (DE3) (TaKaRa BIO, Shiga, Japan). Expression of recombinant protein was induced by adding isopropyl-β-D-thiogalactopyranoside (Nacalai Tesque). The pellets were sonicated for 1 min three times in buffer A [10 mM Tris-HCl (pH 8.0) (Nippon Gene, Tokyo, Japan), 400 mM NaCl (Nacalai Tesque), 5 mM MgCl_2_ (Nacalai Tesque), 0.1 mM phenylmethylsulfonyl fluoride (Nacalai Tesque), 1 mM 2-mercaptoethanol (Nacalai Tesque), and 10% glycerol (Nacalai Tesque)]. After centrifugation of the mixture at 4°C and 17 800 × *g* for 15 min, the supernatants were filtered through a 0.45-µm Millex-HV filter unit (Merck Millipore, Burlington, MA, USA), and the recombinant protein was purified by using an NGC chromatography system (Bio-Rad, Hercules, CA, USA) with a HisTrap HP column (Cytiva, Tokyo, Japan). PspA was eluted with buffer A containing 100–500 mM imidazole (Nacalai Tesque). The eluted protein was loaded into a 30-kDa centrifugal filter unit (Merck Millipore) for concentration and exchange with phosphate-buffered saline (Nacalai Tesque). After concentration of the eluted protein, Triton X-114 non-ionic detergent (Nacalai Tesque) was added to make a 1% concentration. After being vortexed for 30 sec, the protein was kept on ice for 5 min. It was then placed in a water bath at 37°C for 5 min. Finally, after centrifugation at 25°C and 3000 × *g* for 3 min, the supernatant was collected. After this procedure had been performed two times, the concentration of purified protein was measured by using a BCA protein assay kit (Thermo Fisher Scientific, Waltham, MA, USA) and the concentration of endotoxin was measured by using a ToxinSensor Chromogenic LAL Endotoxin Assay Kit (Funakoshi, Tokyo, Japan). The purity of the eluted protein was confirmed in a NuPAGE electrophoresis system (Invitrogen, Carlsbad, CA, USA). This was followed by staining with Coomassie brilliant blue (ATTO, Tokyo, Japan).

### Immunization

Mice were anesthetized with isoflurane (Fujifilm, Tokyo, Japan) and then sublingually immunized with a total volume of 5 μl PBS containing either 5 μg of ovalbumin (OVA) with or without 1 μg of *Alcaligenes* lipid A, 10 µg of lipopolysaccharides from *E. coli* O127:B8 (Sigma-Aldrich, St. Louis, MO, USA), or 10 µg of K3-CpG-ODN (GeneDesign, Osaka, Japan); or 5 μg of PspA with or without 1 μg of *Alcaligenes* lipid A; or 2 μg of CT B subunit (CTB) (Fujifilm) with or without 1 μg of *Alcaligenes* lipid A; or PBS only. To compare administration routes, mice were subcutaneously, intranasally, and intramuscularly immunized with a total volume of 200 µl (subcutaneous or intramuscular administration) or 15 μl (intranasal administration) PBS containing 5 μg of OVA with or without 1 μg of *Alcaligenes* lipid A. After immunization, mice were left forward bending under anesthesia for 30 min. Mice received three immunizations at 1-week intervals. One week after the final immunization, blood was harvested from the mice and kept on ice until centrifugation at 3000 × *g* at 4°C for 10 min. The serum was transferred into a fresh tube. Feces were collected and weighed to make a 100-mg/ml suspension liquid by vortexing for 10 min at 4°C. After centrifugation of the suspension at 4°C and 3000 × *g* for 10 min, the supernatant of the feces was transferred into a fresh tube. Nasal wash fluid was obtained by using 200 µl of PBS via a cut in the trachea. Bronchoalveolar lavage fluid (BALF) was harvested by introducing 1 ml of PBS into the lungs via a cut in the trachea and then flushing and absorbing the fluid five times via a cut in the trachea. All samples were stored at −30°C.

### Detection of antigen-specific antibodies by enzyme-linked immunosorbent assay

Production of OVA-, PspA-, and CT-specific antibodies was detected by enzyme-linked immunosorbent assay (ELISA). Briefly, 96-well immunoplates (Thermo Fisher) were coated with 1 mg/ml of OVA or 5 μg/ml of CT or 5 μg/ml of PspA in PBS at 4°C overnight. After the coating solution was removed, the plates were saturated for 2 h at room temperature with 1% bovine serum albumin (BSA) (Nacalai Tesque) dissolved in PBS. The plates were then rinsed three times with wash buffer [PBS containing 0.05% Tween 20 (Nacalai Tesque)]. Each well then received mouse serum, nasal wash, BALF, or supernatant of the fecal suspension liquid (2-fold serially diluted in PBS containing 0.05% Tween 20 and 1% BSA), and the plates were incubated at room temperature for 2 h. The plates were then again washed three times with wash buffer. Goat anti-mouse IgG, IgG1, IgG2a, IgG2b, IgG3, and IgA antibodies and conjugated with horseradish peroxidase (SouthernBiotech, Birmingham, AL, USA; diluted 1:4000 in PBS containing 1% BSA and 0.05% Tween 20) were added to each well, and the plates were incubated at room temperature for 1 h. The plates were again washed three times with wash buffer. Tetramethylbenzidine peroxidase substrate (SeraCare Life Sciences, Milford, MA, USA) was added to the plates, and the plates were incubated at room temperature for 2 min, after which 0.5 N HCl (Nacalai Tesque) was added to each well. The absorbance of the samples at a wavelength of 450 nm (OD_450_) was measured by using an iMark Microplate Absorbance Reader (Bio-Rad Laboratories, Hercules, CA, USA).

### T cell assay

One week after the final immunization, the submandibular lymph nodes (SMLNs) and spleens from the immunized mice were harvested, homogenized, and then filtered through 100-μm cell strainers (Corning, New York, NY, USA) separately. These single-cell suspensions were treated with 1 ml of red blood cell lysis buffer [10 mM NaHCO_3_ (Nacalai Tesque), 1 mM EDTA-2Na·2H_2_O (Dojindo Molecular Technologies, Kumamoto, Japan), and 0.15 M NH_4_Cl (Nacalai Tesque)] for 1 min at room temperature. CD4^+^ T cells in SMLNs or spleen from immunized mice were purified by using a magnetic cell separation system, anti-mouse CD4 (L3T4) magnetic beads (Miltenyi Biotec, Bergish Gladbach, Germany), and MS columns (Miltenyi Biotec). Purified CD4^+^ T cells were resuspended in RPMI medium (Sigma-Aldrich, St Louis, MO, USA) containing 10% fetal bovine serum (Serana, Brandenburg, Germany), 1 mM sodium pyruvate solution (Nacalai Tesque), 1% penicillin–streptomycin mixed solution (Nacalai Tesque), and 0.1% 2-mercaptoethanol (Gibco, Invitrogen, Waltham, MA, USA) and were seeded at a concentration of 2 × 10^5^ cells/well into 96-well plates (Nunc 96-Well, Nunclon Delta-Treated, U-Shaped-Bottom Microplates, Thermo Fisher Scientific). Each well also received splenic antigen-presenting cells (APCs) (2 × 10^4^ cells/well) from unimmunized mice that had been treated with 30 Gy of ionizing radiation (MBR-1520R-4 x-ray generator, Hitachi, Tokyo, Japan). The purified CD4^+^ T cells mixed with the APCs were incubated in the presence or absence of 1 mg/ml of OVA or 4 μg/ml of PspA at 37°C in 5% CO_2_. After 4 days of incubation, live T cells were counted by using CyQUANT Direct Cell Proliferation Assay Kits (Invitrogen). Cytokines in the supernatant were measured by using a BD CBA (Cytometric Bead Array) Mouse Th1/Th2/Th17 Cytokine Kit (BD Biosciences, San Jose, CA, USA) in accordance with the manufacturer’s instructions and analyzed with a MACSQuant Analyzer (Miltenyi Biotec). Data analysis was performed by using FlowJo 10.0.7 (Tree Star).

### Cell isolation and flow cytometric analysis

The collected tongue tissues were macerated with scissors and incubated with 2.5 mg/ml collagenase (Wako, Tokyo, Japan) in RPMI 1640 medium (Sigma-Aldrich, St. Louis, MO, USA) containing 2% (vol/vol) newborn calf serum (NCS) (Serana, Brandenburg, Germany) with stirring (90 min, 37°C, 5% CO_2_). Cell suspensions were filtered through 100-μm cell strainers (Corning, New York, NY, USA) and centrifuged (10 min, 4°C, 1400 rpm) to collect cells. Cells within the spleens, lymph nodes, and tongues were stained with an anti-CD16/32 monoclonal antibody (dilution ratio, 1:100; TruStain fcX, BioLegend, San Diego, CA, USA) to avoid nonspecific staining. Dead cells were detected with 7-aminoactinomycin D (7-AAD; 1:100; catalog no. 420404, BioLegend) and excluded from the analysis. To prevent non-specific fluorochrome interactions when using multiple Brilliant-series antibodies, Brilliant Stain Buffer Plus (1:5; catalog no. 566385BD, Biosciences) was applied. The following fluorescently labeled monoclonal antibodies were used for flow cytometric analysis: fluorescein 5(6)-isothiocyanate (FITC)-anti-Ly6G (1:100; catalog no. 127606, clone 1A8, BioLegend), FITC-anti-TCRβ (1:100; catalog no. 109206, clone H57-597, BioLegend), phycoerythrin (PE)-anti-I-A^d^ (1:100; catalog no. 553548, clone AMS-32.1, BD Biosciences), PE-anti-PD-1 (1:100; catalog no. 135206, clone 29F.1A12, BioLegend), PE-anti-IgG (1:100; catalog no. 405307, clone Poly4053, BioLegend), allophycocyanin-anti-CD4 (1:100; catalog no. 100516, clone RM4-5, BioLegend), AF647-anti-GL7 (1:100; catalog no. 144606, clone GL7, BioLegend), allophycocyanin-Cyanine (Cy) 7-anti-CD11b (1:100; catalog no. 101226, clone M1/70, BioLegend), allophycocyanin-Cy7-anti-B220 (1:100; catalog no. 103224, clone RA3-6B2, BioLegend), PE-Cy7-anti-CD11c (1:100; catalog no. 117318, clone N418, BioLegend), PE-Cy7-anti-CD3ε (1:100; catalog no. 100320, clone 145-2C11, BioLegend), BV421-anti-TCRβ (1:100; catalog no. 109230, clone H57-597, BioLegend), and Brilliant Ultra Violet 395-anti-CD45 (1:100; catalog no. 564279, clone 30-F11, BD Biosciences). Flow cytometric analysis was conducted on a CytoFLEX LX instrument (Beckman Colter, Brea, CA, USA). Data were analyzed in FlowJo 10.10.0 (Tree Star, Ashland, OR, USA).

### Immunohistogical analysis

Mice were sublingually immunized with 5 μg of biotinylated OVA in 5 μl of PBS to examine the distribution of OVA in sublingual mucosa. At 30 min after administration, tongue tissues were collected from mice, washed with PBS on ice, embedded in Tissue-Tek OCT compound (Sakura Finetek Japan, Tokyo, Japan), and cut into 6-µm sections by using a CM3050 S cryostat (Leica Biosystems, Wetzlar, Germany). Tissue sections of tongues were used for hematoxylin and eosin staining and immunohistological analysis. In the immunohistological analysis, tongue sections were fixed for 1 min at room temperature in prechilled 100% acetone (Nacalai Tesque), followed by fixation for 30 min at 4°C in prechilled 95% ethanol (Nacalai Tesque) and for 1 min at 4°C in prechilled 100% acetone. Tissue sections were washed twice in PBS (each wash for 5 min) and then blocked with 2% NCS (Equitech Bio, Kerrville, TX, USA) in sterile PBS for 30 min at room temperature in an incubation chamber. The tissue sections were then washed in PBS containing 0.1% Tween 20 for 5 min, washed in PBS for 5 min, and then stained with AF546-streptavidin (1:200; Thermo Fisher Scientific) in sterile PBS containing 2% NCS for 30 min at room temperature in an incubation chamber. After being washed twice with PBS for 5 min each time, the tissue sections were stained with 1 µM 4′,6-diamidino-2-phenylindole (DAPI) (AAT Bioquest, Sunnyvale, CA, USA) for 10 min at room temperature in an incubation chamber. Finally, the tissue sections were washed twice in PBS for 5 min each time, mounted in Fluoromount (Diagnostic BioSystems, Pleasanton, CA, USA), and examined under a fluorescence microscope (BZ-9000; Keyence, Osaka, Japan).

### *Streptococcus pneumoniae* culture and infection model

*Streptococcus pneumoniae* Xen10 was cultured overnight in brain–heart infusion broth (Becton, Dickinson and Company, Franklin Lakes, NJ, USA) at 37°C in 5% CO_2_ with no aeration. *Streptococcus pneumoniae* was then collected by centrifugation at 4°C for 15 min at 3000 × *g* and then washed twice with PBS at 4°C for 3 min with centrifugation at 9100 × *g*. One week after the final immunization, mice were nasally challenged with 1.5 × 10^7^ colony-forming units (40 µl per mouse) of *S. pneumoniae* under anesthesia. The survival and body weight of the infected mice were monitored for 14 days. After euthanizing infected mice, lungs were then collected from these mice, and the collected lungs were homogenized for 1 min in 1 ml sterile PBS (Nacalai Tesque). The samples were plated on blood agar (BD Biosciences) coated with kanamycin (100 µg/ml; 40 μl) (Nacalai Tesque). After the sample culture overnight at 37°C, the numbers of bacterial colonies on the blood agar plate were counted.

### CT challenge

One week after the final immunization, mice were fasted for 8 h and orally challenged with 50 μg CT in 200 μl; 16 h after CT challenge as described previously with some modification (21), the volume of liquid in cecum was measured.

### Statistical analysis

Data are presented as means ± 1 SD. Statistical analyses were performed by using Student’s *t*-test and one-way ANOVA with Tukey’s multiple comparison test after ROUT outlier identification (PRISM 10.1.2, GraphPad Software, San Diego, CA, USA). Statistical significance was established at *P* < .05.

## Results

### Sublingual administration of OVA and *Alcaligenes* lipid A induces enhanced antigen-specific humoral immune responses in the respiratory and gastrointestinal tracts through germinal center reactions in the draining lymph nodes and in systemic compartments without excessive inflammation

**Figure 1.**
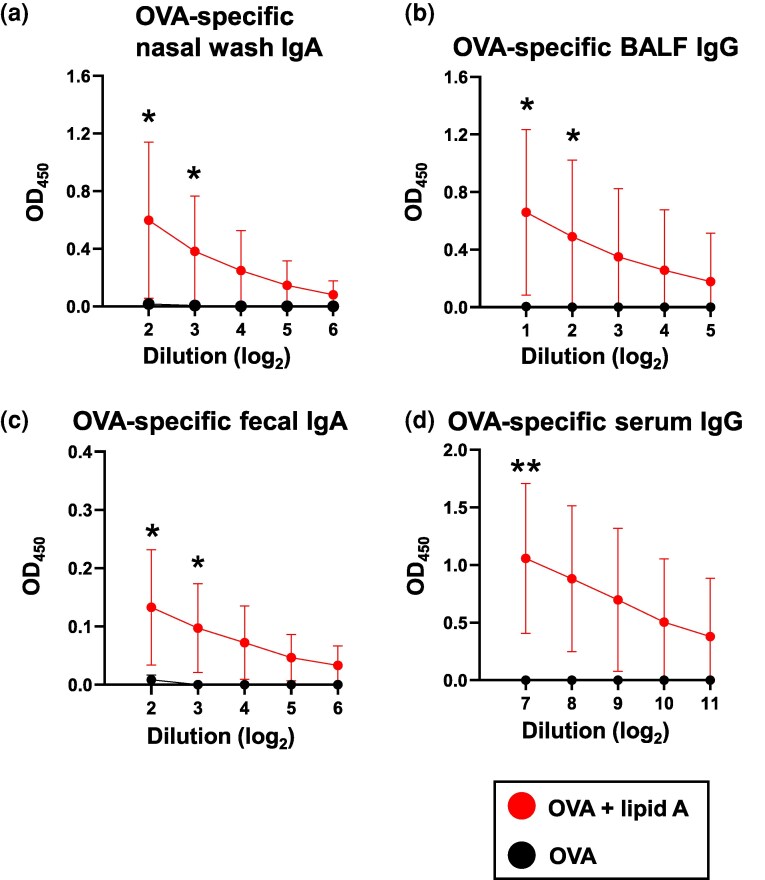
Sublingual administration of *Alcaligenes* lipid A enhances the production of OVA-specific antibodies in both the mucosal and the systemic compartment. Mice were immunized sublingually with OVA with or without *Alcaligenes* lipid A. Nasal wash, BALF, feces, and serum were collected 1 week after the final immunization, and the levels of (a) OVA-specific nasal wash IgA (*n* = 6/group), (b) OVA-specific BALF IgG (*n* = 6/group), (c) OVA-specific fecal IgA (n = 6/group), and (d) OVA-specific serum IgG (experimental group, *n* = 6/group; control group, *n* = 5/group) were measured by ELISA. The results shown are presented as means ± 1 SD. Data are a combination of two independent experiments, and statistical significance was evaluated by using Student’s *t-*test (*P < .05; **P < .01 compared with OVA alone).

To examine whether *Alcaligenes* lipid A can enhance antigen-specific immune responses in both the respiratory tract and the intestinal tract, we sublingually co-immunized mice with *Alcaligenes* lipid A and OVA, a model antigen, to analyze the humoral immune responses. In the respiratory tract, OVA-specific antibody responses, as evidenced by elevated levels of IgA in the nasal wash fluids (Fig. 1a) and IgG in the BALF (Fig. 1b), were enhanced in mice sublingually immunized with OVA plus *Alcaligenes* lipid A, compared with OVA alone. Notably, despite individual variations, a trend toward increased OVA-specific IgA in the BALF was also observed (Figure S1A). Not just the respiratory immune response but also the enhanced OVA-specific fecal IgA response highlighted the effect of *Alcaligenes* lipid A on intestinal immunity as a sublingual adjuvant (Fig. 1c). Moreover, OVA-specific IgG in the serum was enhanced in mice sublingually immunized with OVA alongside *Alcaligenes* lipid A (Fig. 1d). In terms of subclasses, OVA-specific IgG1, IgG2b, and IgG3 were predominantly induced in serum from mice immunized with OVA plus *Alcaligenes* lipid A (Supplementary Figure S1b). Collectively, these findings demonstrated that sublingual immunization using *Alcaligenes* lipid A as an adjuvant robustly enhanced antigen-specific antibody production, not only in the respiratory and intestinal tracts but also systemically. Although the enhancement of antibody production induced by sublingual vaccination was weaker than that induced by intranasal vaccination (Supplementary Figure S2), it is noteworthy that IgA antibody production in the intestinal tract was not observed after intranasal, subcutaneous, or intramuscular administration (Supplementary Figures S2 and S3). In support of these results, we noted that within 30 min after sublingual administration, the antigen was taken up and distributed not only on the sublingual surface but also within the sublingual tissue (Supplementary Figure S4a). This suggests that the rapid absorption of the antigen enables its delivery to immune cells, thereby exerting vaccine effects. Therefore, sublingual vaccination can be considered an effective route of administration for mucosal vaccines.

**Figure 2.**
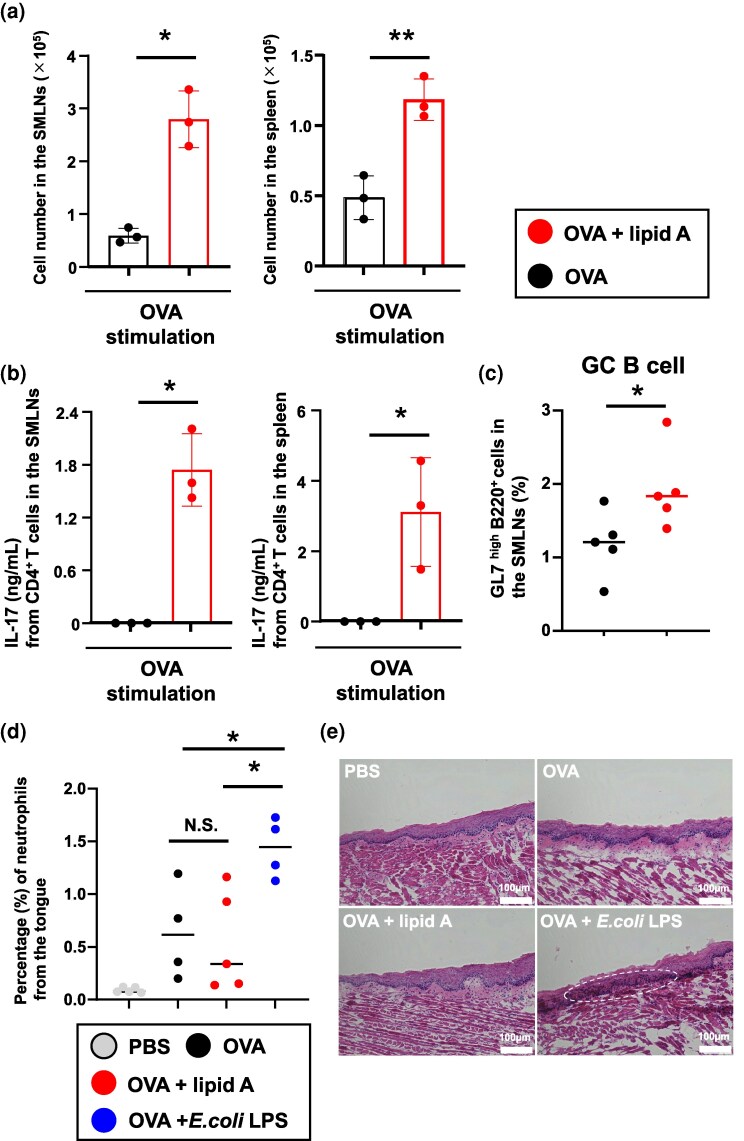
Sublingual administration of *Alcaligenes* lipid A enhances Th17 responses without excessive inflammation at the administration sites. Mice were immunized sublingually with OVA with or without *Alcaligenes* lipid A. CD4^+^ T cells from submandibular lymph nodes (SMLNs) and spleens were collected 1 week after the final immunization, and (a) the number of CD4^+^ T cells in the SMLNs and spleen (*n* = 3/group) was measured by using CyQUANT Direct Cell Proliferation Assay Kits. In addition, (b) the levels of IL-17A from OVA-stimulated T cells from SMLNs and spleen (*n* = 3/group) were measured by using a cytometric bead array kit. The results are presented as means ± 1 SD. Data are a combination of two independent experiments, and statistical significance was evaluated by using Student’s *t-*test (*P < .05; **P < .01 compared with OVA alone). (c, d) Flow cytometric quantification of immune cells isolated from (c) SMLNs and (d) tongue. Gating strategy: germinal center (GC) B cells, CD45⁺ GL7^high^ B220⁺; neutrophils, CD45⁺ CD11b⁺ Ly6G⁺ cells. Data are from two independent experiments; each point represents an individual mouse. Statistical significance was evaluated by using Student’s *t-*test (*P < .05; **P < .01 compared with OVA alone). (e) Histological analysis of frozen tongue sections. The white dotted line indicates an accumulation of inflammatory cells. Scale bars = 100 μm.

Furthermore, we explored whether *Alcaligenes* lipid A induced Th17 responses through sublingual immunization, as we previously reported for nasal or systemic immunization (16). Upon *in vitro* restimulation with APCs plus OVA, CD4^+^ T cells from both the draining lymph nodes (e.g. SMLNs) and spleen of mice immunized with OVA plus *Alcaligenes* lipid A proliferated (Fig. 2a) and secreted significantly higher levels of IL-17A than those of naïve mice or mice immunized with OVA alone (Fig. 2b). In addition, we sublingually immunized the mice with OVA plus CpG-DNA or *Alcaligenes*-derived lipid A and examined cytokine production in harvested T cells, focusing on IL-6 and IL-10 as indicators. We found that *Alcaligenes* lipid A more strongly induced IL-10 and IL-6 production by splenic CD4⁺ T cells than did the addition of CpG-DNA (Supplementary Figure S4b and c). To investigate the mechanisms of immune activation induced by sublingual administration of *Alcaligenes* lipid A, flow cytometric analyses were performed on various immune cell populations. Whereas neither DCs, neutrophils, nor follicular helper T cells showed any changes (Supplementary Figure S4d), the proportion of germinal center (GC) B cells was increased in the *Alcaligenes* lipid A group (Fig. 2c). These results suggest that *Alcaligenes* lipid A, as a sublingual adjuvant, enhances antigen-specific IgA production at mucosal sites by promoting GC B cell responses and Th17 cell responses at mucosal sites.

We further examined whether sublingual administration of lipid A from *Alcaligenes* induces excessive inflammation at the administration sites. Flow cytometry and histological analyses of tongue tissue after the administration of each adjuvant revealed that *E. coli* LPS—but not *Alcaligenes* lipid A—induced increases in neutrophil numbers and the accumulation of inflammatory cells (Fig. 2d and e). Therefore, as a sublingual adjuvant, *Alcaligenes* lipid A appears to enhance vaccine efficacy but avoids excessive inflammation and local tissue damage, suggesting a favorable safety profile.

### Sublingual co-administration of *Alcaligenes* lipid A enhances CTB-specific mucosal and systemic humoral immune responses

**Figure 3.**
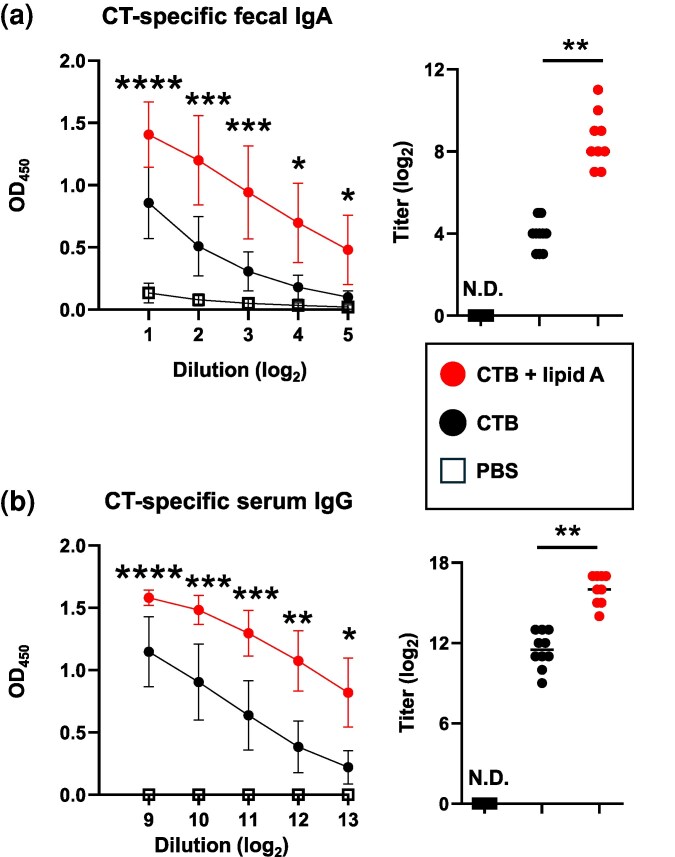
Sublingual administration of *Alcaligenes* lipid A enhances the production of CTB-specific antibodies. Mice were immunized sublingually with PBS or CTB with or without *Alcaligenes* lipid A. Feces and serum were collected 1 week after the final immunization, and the levels of (a) CTB-specific fecal IgA (*n* = 10/group) and (b) CTB-specific serum IgG (positive experimental group, *n* = 9/group; negative experimental group and control group, *n* = 10/group) were measured by ELISA. The results shown are presented as means ± 1 SD. Data are a combination of two independent experiments, and statistical significance was evaluated by using one-way ANOVA (*P < .05; **P < .01; ***P < .001; ****P < .0001; asterisks indicate a significant difference between group CTB plus lipid A and group CTB).

Having verified that sublingually administered *Alcaligenes* lipid A could enhance antigen-specific antibody production in the intestinal tract, we next explored its potential as an adjuvant for sublingual vaccines against intestinal diseases. CTB has been employed as a model vaccine against *Vibrio cholerae* (22). Unlike mice given OVA, mice immunized with CTB alone exhibited a measurable CT-specific IgA response in the feces (Fig. 3a) and a specific IgG response in the serum (Fig. 3b) since CTB has been shown to possess potent mucosal immunogenicity (23). Notably, co-administration of CTB with *Alcaligenes* lipid A resulted in further elevation of the IgA response in the feces (Fig. 3a) and the IgG response in the serum (Fig. 3b). In terms of subclasses, CT-specific IgG1, IgG2b, and IgG3 were predominantly induced in serum from mice immunized with CTB plus *Alcaligenes* lipid A (Supplementary Figure S5). These findings suggest that *Alcaligenes* lipid A is a potent enhancer of antigen-specific antibody responses against intestinal pathogen-derived toxins.

### Sublingual administration of CTB and *Alcaligenes* lipid A ameliorates CT-induced diarrhea

**Figure 4.**
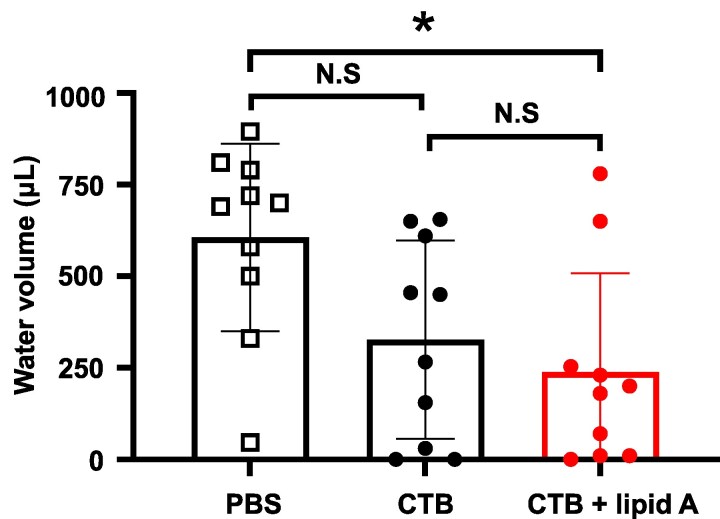
Enhancement of CT-specific immune responses by sublingual administration of *Alcaligenes* lipid A suppresses CT-induced diarrhea. Mice were immunized sublingually with PBS or CTB with or without *Alcaligenes* lipid A. One week after the final immunization, the mice were fasted and orally challenged with a high dose of CT. The cecal liquid volume was measured (*n* = 10/group). The titer was defined as the lowest serum dilution for which OD_450_ exceeded 0.1 (N.D., not detected). Data are a combination of two independent experiments, and statistical significance was evaluated by using one-way ANOVA (*P < .05).

In light of the above results, we sought to determine whether sublingual administration of CTB with *Alcaligenes* lipid A could confer protective immunity against CT challenge. One week after final immunization, mice were orally challenged with CT, and the water content of the cecum was collected and measured to assess signs of diarrhea. As anticipated from the data on intestinal IgA production, mice immunized with CTB alone exhibited a trend toward reduced liquid content in the cecum, but the difference was not statistically significant, because CTB possesses high antigenicity. In contrast, mice immunized with CTB with *Alcaligenes* lipid A demonstrated a significant reduction in cecal liquid content compared with unimmunized controls (Fig. 4). These results indicate that *Alcaligenes* lipid A serves as a potent adjuvant for sublingual vaccines, offering protection against intestinal diarrheal infections.

### Sublingual co-administration of *Alcaligenes* lipid A enhances PspA-specific mucosal and systemic humoral immune responses

**Figure 5.**
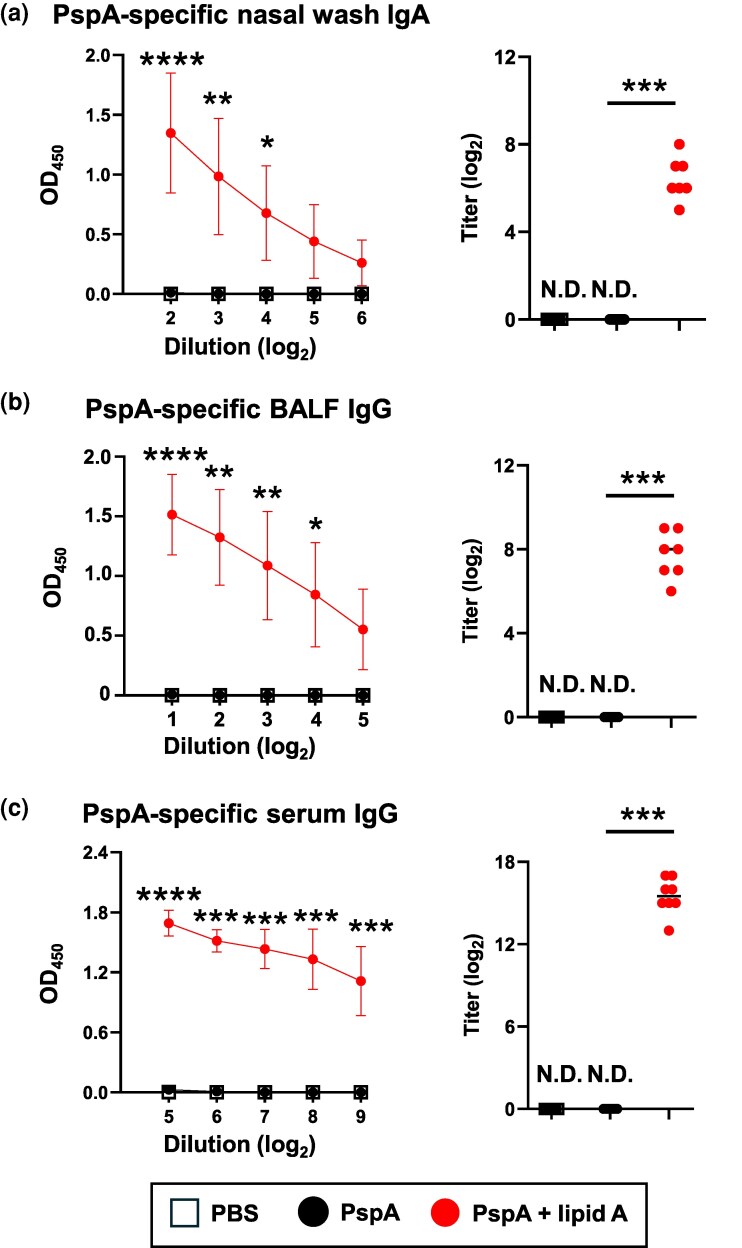
Sublingual administration of *Alcaligenes* lipid A enhances the production of PspA-specific antibodies. Mice were immunized sublingually with PBS or PspA with or without *Alcaligenes* lipid A. Nasal wash, BALF, and serum were collected 1 week after the final immunization, and the levels of (a) PspA-specific nasal wash IgA (positive experimental group, *n* = 7/group; negative experimental group, *n* = 8/group, control group, *n* = 6/group), (b) PspA-specific BALF IgG (positive experimental group, *n* = 8/group; negative experimental group, *n* = 6/group, control group, *n* = 8/group), and (c) PspA-specific serum IgG (positive experimental group, *n* = 8/group; negative experimental group and control group, *n* = 6/group) were measured by ELISA. The results are presented as means ± 1 SD. The titer was defined as the lowest serum dilution for which OD_450_ exceeded 0.1 (N.D., not detected). Data are a combination of two independent experiments, and statistical significance was evaluated by using one-way ANOVA (*P < .05; **P < .01; ***P < .001; ****P < .0001; asterisks represent a significant difference between group PspA plus lipid A and group PspA).

To further assess the potential of *Alcaligenes* lipid A as a sublingual adjuvant for respiratory infections, we evaluated its effects by using PspA, an antigen that is broadly expressed across most serotypes of *S. pneumoniae* and is known for its protective role against pneumococcal infection in mice (24, 25). In mice immunized with PspA alone, no PspA-specific antibody response was detected in any of the samples measured. In contrast, sublingual immunization with PspA and *Alcaligenes* lipid A resulted in the robust enhancement of PspA-specific IgA antibodies in the nasal cavity (Fig. 5a), as well as increased PspA-specific IgG responses in both the BALF and the serum and increased PspA-specific IgA responses in the BALF (Fig. 5b and c and Supplementary Figure S6a). In terms of subclasses, PspA-specific IgG1, IgG2b, and IgG3 were predominantly induced in serum from mice immunized with PspA plus *Alcaligenes* lipid A (Supplementary Figure S6b). These findings show that *Alcaligenes* lipid A effectively amplifies antigen-specific humoral immune responses against respiratory infectious diseases.

### Sublingual co-administration of *Alcaligenes* lipid A enhances PspA-specific Th17 responses

**Figure 6.**
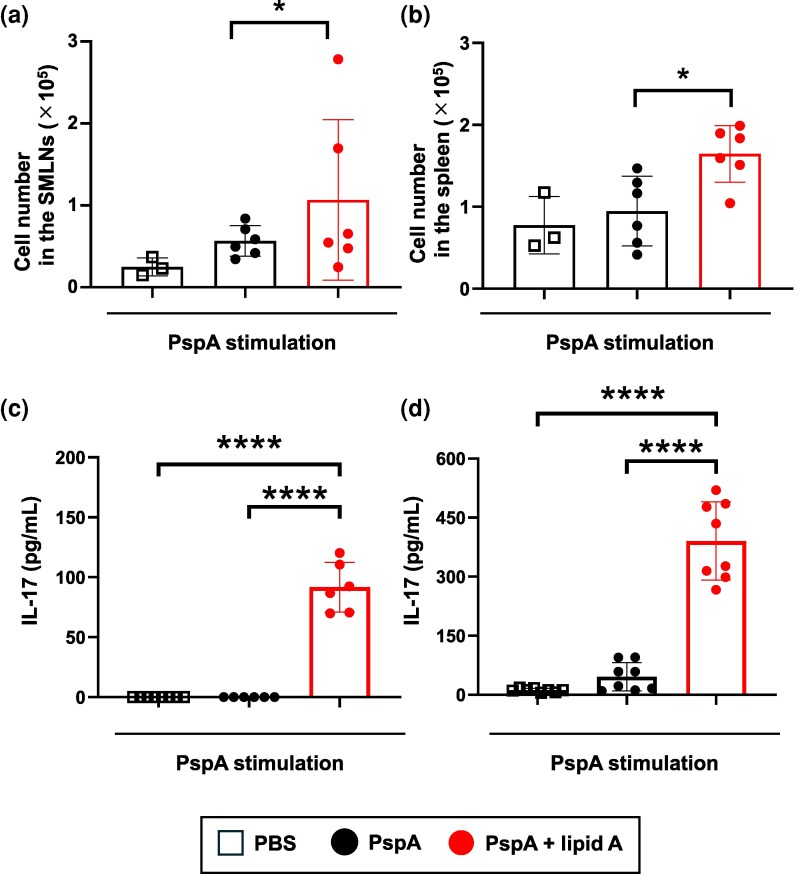
Sublingual administration of *Alcaligenes* lipid A enhances CD4^+^ T cell proliferation and PspA-specific Th17 responses. Mice were immunized sublingually with PBS or PspA with or without *Alcaligenes* lipid A. CD4^+^ T cells from SMLNs and spleens were collected 1 week after the final immunization, and the number of CD4^+^ T cells in (a) SMLNs and (b) spleen were measured by using CyQUANT Direct Cell Proliferation Assay Kits (positive experimental group, *n* = 6/group; negative experimental group, *n* = 6/group, control group, *n* = 3/group). Representative data from two independent experiments with similar results are shown. In addition, the levels of IL-17A from PspA-stimulated T cells of (c) SMLNs (positive experimental group, *n* = 6/group; negative experimental group, *n* = 7/group, control group, *n* = 8/group) and (d) spleen (*n* = 8/group) were measured by using a cytometric bead array kit. The results are presented as means ± 1 SD. Data are a combination of two independent experiments, and statistical significance was evaluated by using one-way ANOVA (****P < .0001).

Beyond humoral immunity, Th17 responses are critical in defending the host against extracellular bacteria such as *S. pneumoniae* (26). We therefore explored whether *Alcaligenes* lipid A could induce Th17 responses through sublingual immunization, as we previously reported in nasal or systemic immunization (16). To test this possibility, CD4^+^ T cells were isolated from SMLNs—primary sites for the induction of antigen-immune responses to sublingual vaccine antigens (27) (Fig. 6a and c and Supplementary Figure S7a)—and the spleen (Fig. 6b and d). Upon *in vitro* restimulation with APCs plus PspA, CD4^+^ T cells from both tissues of mice immunized with PspA plus *Alcaligenes* lipid A proliferated and secreted significantly higher levels of IL-17A than those of naïve mice or mice immunized with PspA alone. In contrast, none of the treatment groups showed any changes in IFN-γ or IL-4 production (Supplementary Figure S7b–e). These findings suggest that *Alcaligenes* lipid A enhances antigen-specific Th17 immune responses, offering increased protection against respiratory infectious diseases.

### Sublingual immunization with PspA and *Alcaligenes* lipid A protects against respiratory infection with *S. pneumoniae*

**Figure 7.**
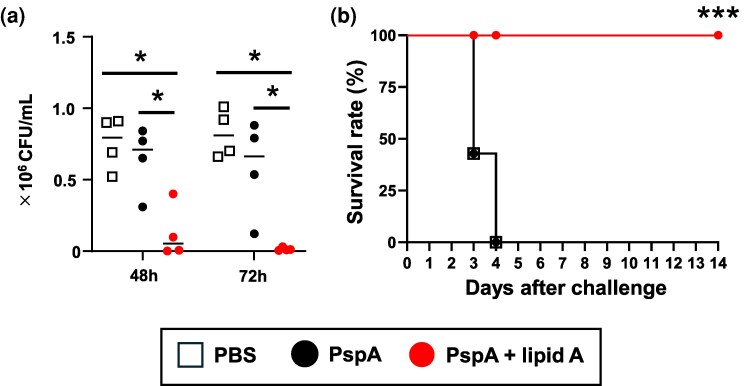
*Alcaligenes* lipid A-enhanced PspA-specific immune responses protect mice from *Streptococcus pneumoniae* infection through bacterial clearance from the lungs. Mice were immunized sublingually with PBS or PspA with or without *Alcaligenes* lipid A. One week after the final immunization and 1 day after retrieval of samples through partial blood collection, mice were nasally challenged with a lethal dose of *S. pneumoniae*. At 48 and 72 h after infection, lungs were collected and (a) *S. pneumoniae* organisms (CFU/ml) were counted (*n* = 4/group). Representative data from two independent experiments with similar results are shown, and statistical significance was evaluated by using one-way ANOVA (*P < .05). (b) Survival rates were calculated each day for 2 weeks (*n* = 7/group). Data are a combination of two independent experiments, and statistical significance was evaluated by using Kaplan–Meier survival analysis (***P < .001; asterisks represent a significant difference between group PspA plus lipid A and group PspA or group PBS).

Our findings regarding sublingually induced humoral and Th17 responses prompted us to investigate whether sublingual immunization with PspA plus *Alcaligenes* lipid A could provide protective immunity against *S. pneumoniae* infection. One week after the last immunization, mice were challenged with *S. pneumoniae* via the respiratory route, and the *S. pneumoniae* organisms in the lungs were counted. In the groups given either PBS or PspA only, the lungs contained numerous *S. pneumoniae* at both 48 and 72 h after infection, whereas pneumococcal counts in lungs from the *Alcaligenes* lipid A group remained low at both time points (Fig. 7a). In addition, we monitored body weight and survival rate for 14 days. The body weights of naïve mice and mice immunized with PspA alone declined rapidly (Supplementary Figure S8), and all mice succumbed by Day 3 (Fig. 7b). In contrast, mice immunized with PspA plus *Alcaligenes* lipid A showed rapid recovery of body weight from Day 2 (Supplementary Figure S8) and all survived (Fig. 7b). These results demonstrate that *Alcaligenes* lipid A is an effective adjuvant for sublingual vaccines, providing effective protection against respiratory infection by *S. pneumoniae* in mice.

## Discussion

We examined the potential of *Alcaligenes* lipid A as an adjuvant for sublingual vaccines, and its immunological effects, by using models of respiratory and intestinal infection. Specifically, *Alcaligenes* lipid A enhanced both PspA- and CT-specific humoral responses in the respiratory and intestinal tracts, as well as in the systemic compartment, thereby providing protection against both mucosal infections. LPS is subject to immunological tolerance in the intestine because of continuous exposure to it in commensals and foods (28). Therefore, sublingual administration is expected to circumvent the insufficient adjuvant activity of *Alcaligenes* lipid A in oral vaccines to induce intestinal immune responses.

Whereas subcutaneous and intramuscular administration primarily enhanced OVA-specific IgG production in serum only, sublingual and intranasal administration also induced antigen-specific IgA production at mucosal surfaces (Supplementary Figure S3). Notably, sublingual administration elicited IgA responses in both the gastrointestinal and respiratory tracts (Supplementary Figure S3). The sublingual route allows antigens to avoid degradation in the gastrointestinal tract, enabling effective immune responses even at low doses. In addition, the thin non-keratinized epithelium and rich blood supply of the sublingual mucosa provide high permeability, facilitating efficient uptake of both antigen and adjuvant, which likely contributes to the advantages of sublingual vaccination. *Alcaligenes* lipid A possesses unique structural features that distinguish it from *E. coli*-derived lipid A in the number of acyl chains and the degree of phosphorylation as well as differences in functional groups (17). Consequently, *Alcaligenes* lipid A exhibits poor water solubility and limited dispersibility in aqueous environments, which may affect its absorption through the sublingual mucosa. Although chemical or fluorescent modification could enable biodistribution analysis of *Alcaligenes* lipid A, such approaches remain technically and economically challenging. Therefore, the lack of direct evidence for lipid A uptake into the sublingual mucosa represents a limitation of the present study. To improve delivery efficacy, combining *Alcaligenes* lipid A with suitable drug delivery systems (DDS) would be advantageous. Among various strategies, mucoadhesive films and nanoparticle formulations appear particularly promising. Mucoadhesive films can prolong the residence time of the vaccine formulation on the sublingual surface, thereby enhancing the uptake of both antigen and adjuvant. Furthermore, nanoparticle-based systems—such as lipid nanoparticles (LNPs) or biodegradable polymeric nanoparticles (e.g. PLGA)—can improve the dispersibility and mucosal permeability of *Alcaligenes* lipid A. Although the fluorescence signal of biotin-labeled OVA was widely distributed within the sublingual tissue (Supplementary Figure S4a), this likely reflects diffusion and uptake of antigen into the superficial epithelial and submucosal layers rather than massive protein absorption. Even limited antigen penetration into the sublingual mucosa is sufficient to activate local APCs and initiate immune responses, as reported previously (6). These results and information indicate that sublingual vaccination is an effective mucosal vaccination strategy capable of inducing IgA production across multiple mucosal tissues.

A representative approach that utilizes sublingual administration is sublingual immunotherapy (SLIT). SLIT with allergens, such as house dust mite antigens, primarily aims to modulate helper T cell balance, promoting regulatory T cell responses and shifting the Th1/Th2 equilibrium to reduce allergic inflammation (29). In contrast, sublingual vaccination with antigens combined with adjuvants, such as *Alcaligenes* lipid A, focuses on amplifying specific immune responses, including Th17- and GC B cell-mediated pathways, to enhance mucosal and systemic antibody production (Figs 1 and 2). Although SLIT promotes immune tolerance and long-term regulation, adjuvant-enhanced sublingual immunization is designed to actively boost protective immunity without inducing excessive inflammation. These distinctions highlight the potential to tailor sublingual strategies either for allergy management or for robust vaccine-induced immunity, depending on the desired immunological outcome.

Our previous studies reported that *Alcaligenes*-driven LPS and lipid A could activate DCs by enhancing the expression of major histocompatibility complex (MHC) class II and costimulatory molecules (e.g. CD40, CD80, and CD86), as well as the secretion of IL-6 and IL-23 (16, 18). These molecules are required to activate T cells (30), and IL-6 and IL-23 are keys for Th17-cell differentiation, survival, and proliferation (31). In our study, we found that *Alcaligenes* lipid A induced IL-10 and IL-6 production by splenic CD4⁺ T cells more strongly than did the addition of CpG-DNA (Supplementary Figure S4b and c). IL-6 promotes effector responses by driving Th17 differentiation, suppressing Treg development, and supporting follicular helper T-mediated B cell help, leading to robust antibody production and memory formation (32–34). In contrast, IL-10 secreted by effector and regulatory T cells functions as a negative regulator, suppressing excessive mucosal inflammation and APC activity, but potentially limiting the magnitude of effector and humoral responses (35 , 36). Collectively, these findings suggest that a balance between IL-6 and IL-10 is critical for mucosal vaccine efficacy. On the basis of the results of this study, the induction of both IL-10 and IL-6 suggests that *Alcaligenes* lipid A may serve as a useful sublingual adjuvant that can activate mucosal immunity while simultaneously regulating excessive inflammation.

In addition, sublingual immunization promotes the migration of immune cells to distant mucosal sites such as the intestinal tract (6). In this regard, expression of CCL19 and CCL21 is significantly enhanced in the cervical lymph nodes, a process thought to be regulated by epithelial or stromal cells rather than by DCs or macrophages (37). This is accompanied by the activation of antigen-specific CD4^+^ T and B cell responses induced by DCs (38). After activation, DCs and T and B cells are recruited to distant mucosal tissues (e.g. nasopharynx-associated lymphoid tissues and Peyer’s patches) through the axis of CCL19/CCL21-CCR7 (37, 39), resulting in protection from both respiratory and intestinal infection. In addition, *Alcaligenes* lipid A upregulates the expression of CCL2 on stromal cells and CCL3 on CD45^+^ immune cells, enhancing the recruitment and infiltration of DCs in nasal tissues (40). These findings, together with our previous findings that *Alcaligenes*-driven LPS and lipid A can activate DCs, with enhanced T cell responses and antibody production, suggest that DCs that are directly activated by *Alcaligenes* lipid A and indirectly activated by *Alcaligenes* lipid A-influenced stromal cells play an important role in enhancing protective immune responses during sublingual administration.

Only a few adjuvants for mucosal vaccines have been examined. Monophosphoryl lipid A (MPLA) is an adjuvant that is used clinically and acts as a TLR4 agonist (41, 42), such as in human papillomavirus vaccines. We previously compared the effects and potential mechanism of MPLA and *Alcaligenes* lipid A as mucosal adjuvants. In mice, intranasal administration of *Alcaligenes* lipid A resulted in increased production of antigen-specific IgA antibodies in nasal wash fluids, whereas no immune response was induced by MPLA (40). Mechanistically, MPLA has been described as a TICAM1- (TRIF-) biased TLR4 agonist (41), whereas our previous study demonstrated that *Alcaligenes* lipid A could activate DCs through both the MyD88 and the TRIF signaling pathways. Enhanced IL-6 secretion and the expression of costimulatory molecules were linked to the MyD88 pathway (18). These findings further illustrate how *Alcaligenes* lipid A activates DCs through sublingual administration.

As a safety consideration, we previously demonstrated that nasal immunization with PspA combined with *Alcaligenes* lipid A did not recruit neutrophils under normal conditions but effectively recruited neutrophils upon *S. pneumoniae* infection, preventing bacterial growth in the lung tissues (19). In other words, intranasal immunization using *Alcaligenes* lipid A as an adjuvant does not induce inflammation characterized by neutrophil increase but can trigger protection from neutrophil-mediated infection, specifically when a pathogen matching the vaccine invades, making it a highly safe vaccine adjuvant. Furthermore, whereas nasal administration is known to lead to antigen accumulation in the olfactory bulbs and brain, sublingual administration is considered a safer alternative (38 ). CT, which can enhance immune responses in both the nasal and intestinal mucosae, often induces excessive inflammation, such as the recruitment of extra neutrophils (19). In contrast, *Alcaligenes* lipid A demonstrates greater safety, as reflected by stable body weight and body temperature (16). In our study, unlike *E. coli* LPS, sublingual administration of *Alcaligenes* lipid A did not elicit neutrophil infiltration or inflammatory cell accumulation in the tongue (Fig. 2d and e). These findings suggest that *Alcaligenes* lipid A functions as an effective sublingual adjuvant, with minimal local inflammation and tissue injury, thereby supporting its favorable safety profile.

The activity of lipid A/LPS as a TLR4 agonist is closely related to its chemical structure (43). We have compared the structure of *Alcaligenes*-driven lipid A and *E. coli*-driven lipid A and have identified differences, including differences in the length, portion, and modification of acyl carbon chains (17). In addition, a major structural difference between MPLA and *Alcaligenes* lipid A lies in the numbers of phosphoryl groups, resulting in inefficient dimerization of the TLR4-MD-2 complex by MPLA, thus decreasing the activation of MyD88 (18). Although further studies are required to support our theory, we suggest that the unique structure of *Alcaligenes* lipid A might contribute to its application as a suitable sublingual vaccine adjuvant.

Although sublingual vaccination offers several advantages, including non-invasive administration and efficient mucosal immune induction, several inherent limitations should be considered. First, the effective antigen dose is generally lower than in parenteral routes, which may limit the magnitude of systemic immune responses and would require careful optimization of antigen and adjuvant combinations. Second, antigen stability can be a concern, given that proteins or peptides may degrade under storage conditions or within the oral cavity, potentially reducing vaccine efficacy. Third, whether the immune response is sustained after sublingual vaccination has not yet been established, with scant data on long-term antibody persistence and memory T cell maintenance compared with the information available for intramuscular or subcutaneous immunization. Finally, although the local and systemic side effects of sublingual vaccination are generally mild, the potential for local irritation or hypersensitivity reactions must be monitored, particularly when novel adjuvants are provided. Addressing these limitations is crucial to achieve broad clinical application of sublingual vaccines.

In conclusion, *Alcaligenes* lipid A could exert a role as a sublingual vaccine adjuvant to induce strong antigen-specific Th17 responses and antibody production in both the respiratory tract and the intestinal tract, resulting in protection against extracellular bacteria and toxins. We have verified that *Alcaligenes* lipid A is an effective and safe adjuvant that can enhance antigen-specific antibody responses systemically and at several mucosal sites. In addition, sublingual administration could overcome the limitations and safety issues of oral or nasal administration. Besides, the sublingual administration as a new route of vaccine administration extended the application of *Alcaligenes* lipid A as a suitable adjuvant. We can therefore conclude that sublingual vaccines have superior suitability for the application of *Alcaligenes* lipid A as a mucosal adjuvant. Currently, non-clinical trials to verify the effect of *Alcaligenes* lipid A have been performed. In the future, the adjuvanticity and safety of *Alcaligenes* lipid A and of sublingual vaccines in humans need to be examined.

## Supplementary Material

dxaf066_Supplementary_Data
