# Supplementary material for: Alcaligenes lipid A as a sublingual adjuvant to augment protective immune responses in the respiratory and gastrointestinal tracts
Source: Int Immunol. 2025 Nov 26;38(4):264–77. doi: 10.1093/intimm/dxaf066 (PMC13042242; doi:10.1093/intimm/dxaf066)
Supplement: dxaf066_Supplementary_Data [file dxaf066_supplementary_data.pdf]

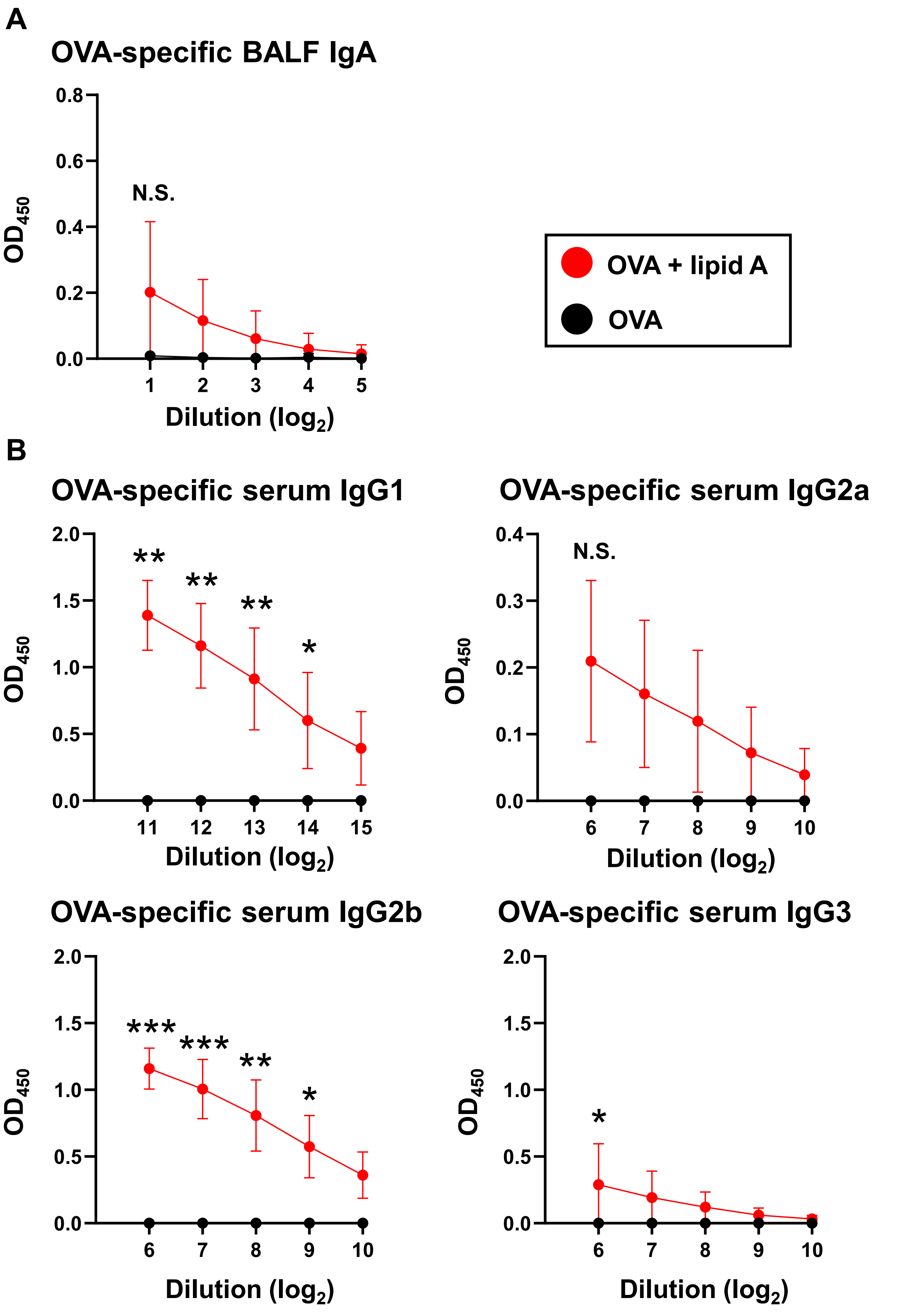

A
OVA-specific BALF IgA
OD450
0.8
0.6
0.4
0.2
0.0
N.S.
1
2
3
4
5
Dilution (log2)
OVA + lipid A
OVA
B
OVA-specific serum IgG1
2.0
1.5
1.0
0.5
0.0
**
**
**
*
11
12
13
14
15
OVA-specific serum IgG2a
0.4
0.3
0.2
0.1
0.0
N.S.
6
7
8
9
10
OVA-specific serum IgG2b
***
***
**
*
OVA-specific serum IgG3
*

Fig. S1

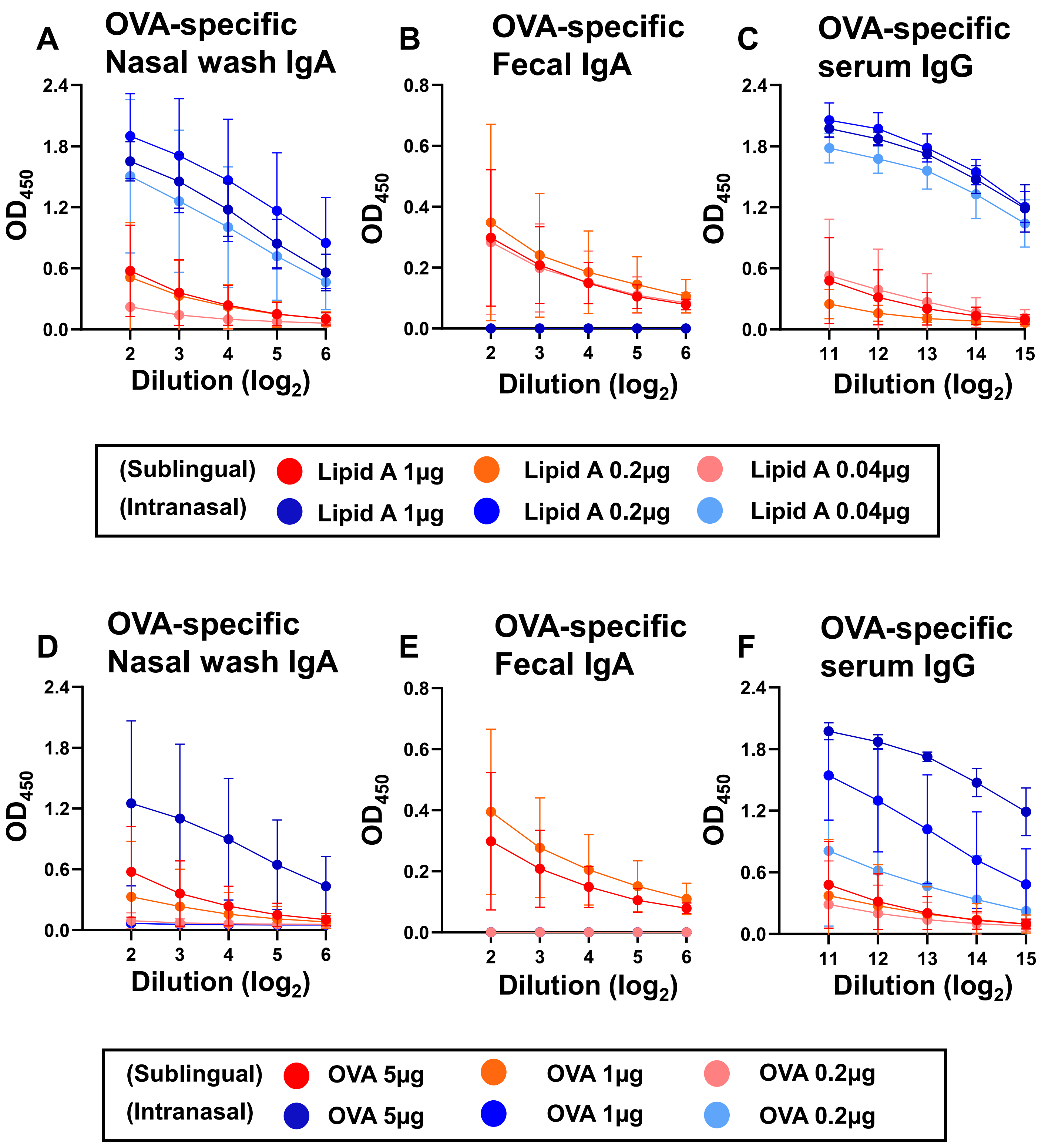
A
OVA-specific Nasal wash IgA
B
OVA-specific Fecal IgA
C
OVA-specific serum IgG
OD450
Dilution (log2)
(Sublingual) Lipid A 1μg Lipid A 0.2μg Lipid A 0.04μg
(Intranasal) Lipid A 1μg Lipid A 0.2μg Lipid A 0.04μg
D
OVA-specific Nasal wash IgA
E
OVA-specific Fecal IgA
F
OVA-specific serum IgG
(Sublingual) OVA 5μg OVA 1μg OVA 0.2μg
(Intranasal) OVA 5μg OVA 1μg OVA 0.2μg

Fig. S2

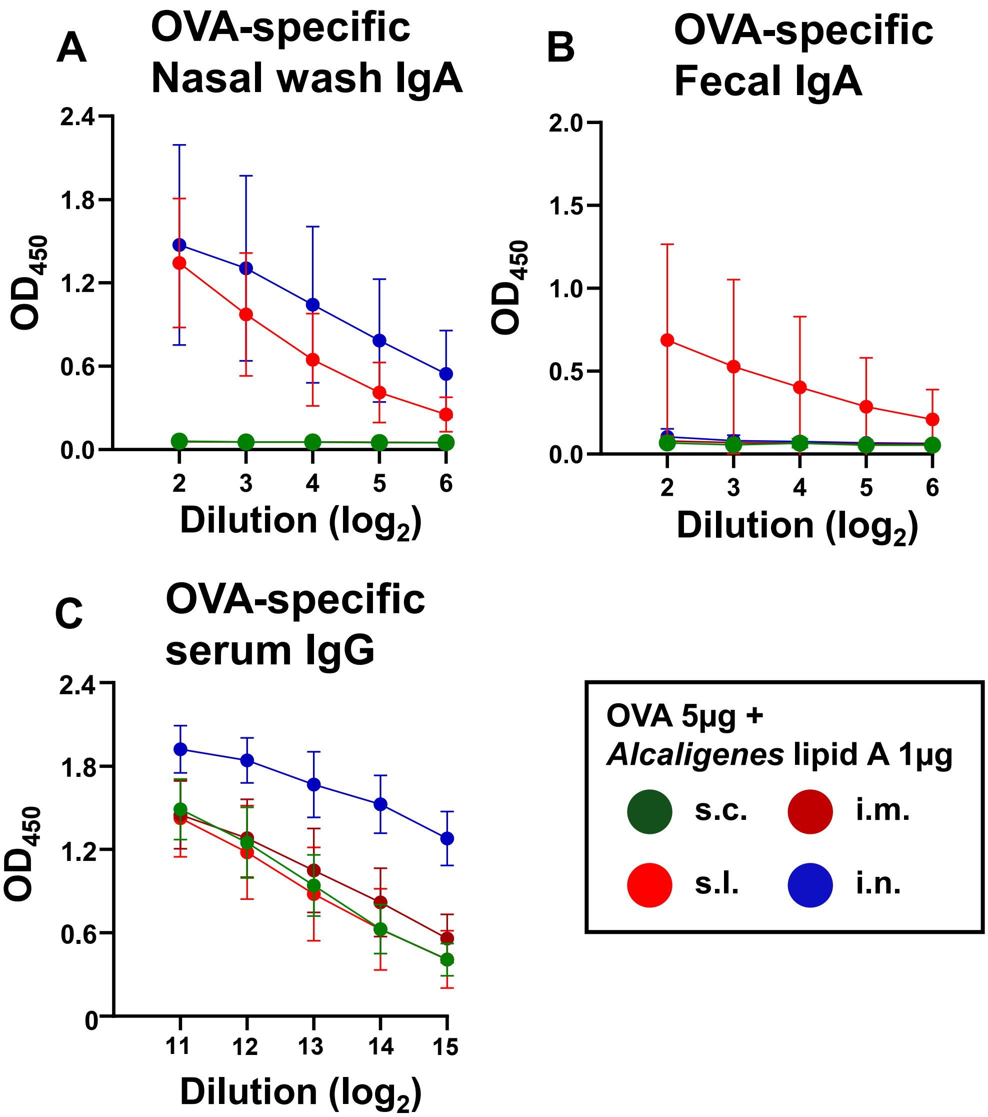
A
OVA-specific
Nasal wash IgA
OD450
2.4
1.8
1.2
0.6
0.0
2
3
4
5
6
Dilution (log2)
B
OVA-specific
Fecal IgA
OD450
2.0
1.5
1.0
0.5
0.0
2
3
4
5
6
Dilution (log2)
C
OVA-specific
serum IgG
OD450
2.4
1.8
1.2
0.6
0
11
12
13
14
15
Dilution (log2)
OVA 5μg +
Alcaligenes lipid A 1μg
s.c.
i.m.
s.l.
i.n.

Fig. S3

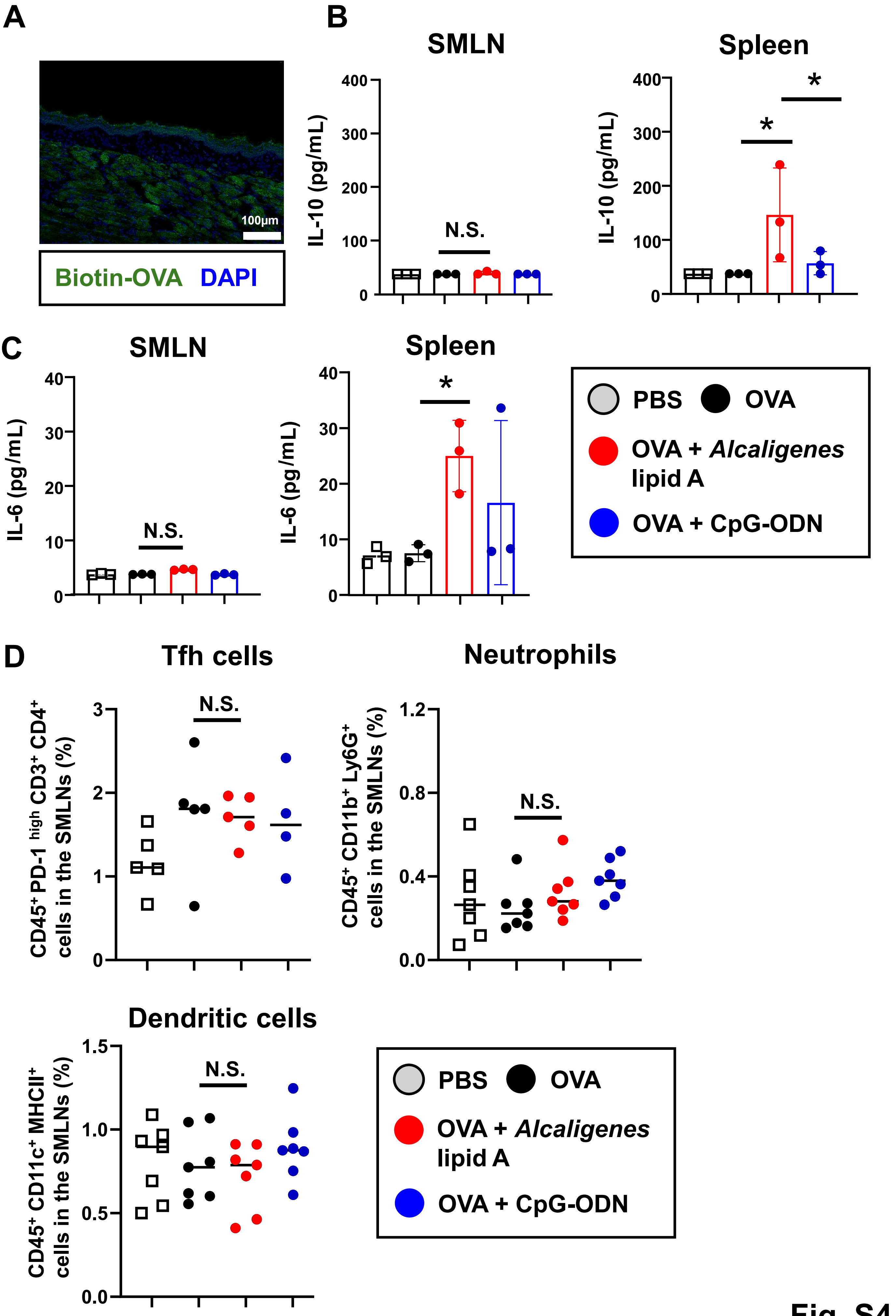

A
100μm
Biotin-OVA DAPI
B
SMLN
Spleen
IL-10 (pg/mL)
400
300
200
100
0
N.S.
*
*
C
SMLN
Spleen
IL-6 (pg/mL)
40
30
20
10
0
N.S.
*
PBS OVA
OVA + Alcaligenes lipid A
OVA + CpG-ODN
D
Tfh cells
CD45⁺ PD-1 high CD3⁺ CD4⁺ cells in the SMLNs (%)
3
2
1
0
N.S.
Neutrophils
CD45⁺ CD11b⁺ Ly6G⁺ cells in the SMLNs (%)
1.2
0.8
0.4
0.0
N.S.
Dendritic cells
CD45⁺ CD11c⁺ MHCII⁺ cells in the SMLNs (%)
1.5
1.0
0.5
0.0
N.S.
PBS OVA
OVA + Alcaligenes lipid A
OVA + CpG-ODN

Fig. S4

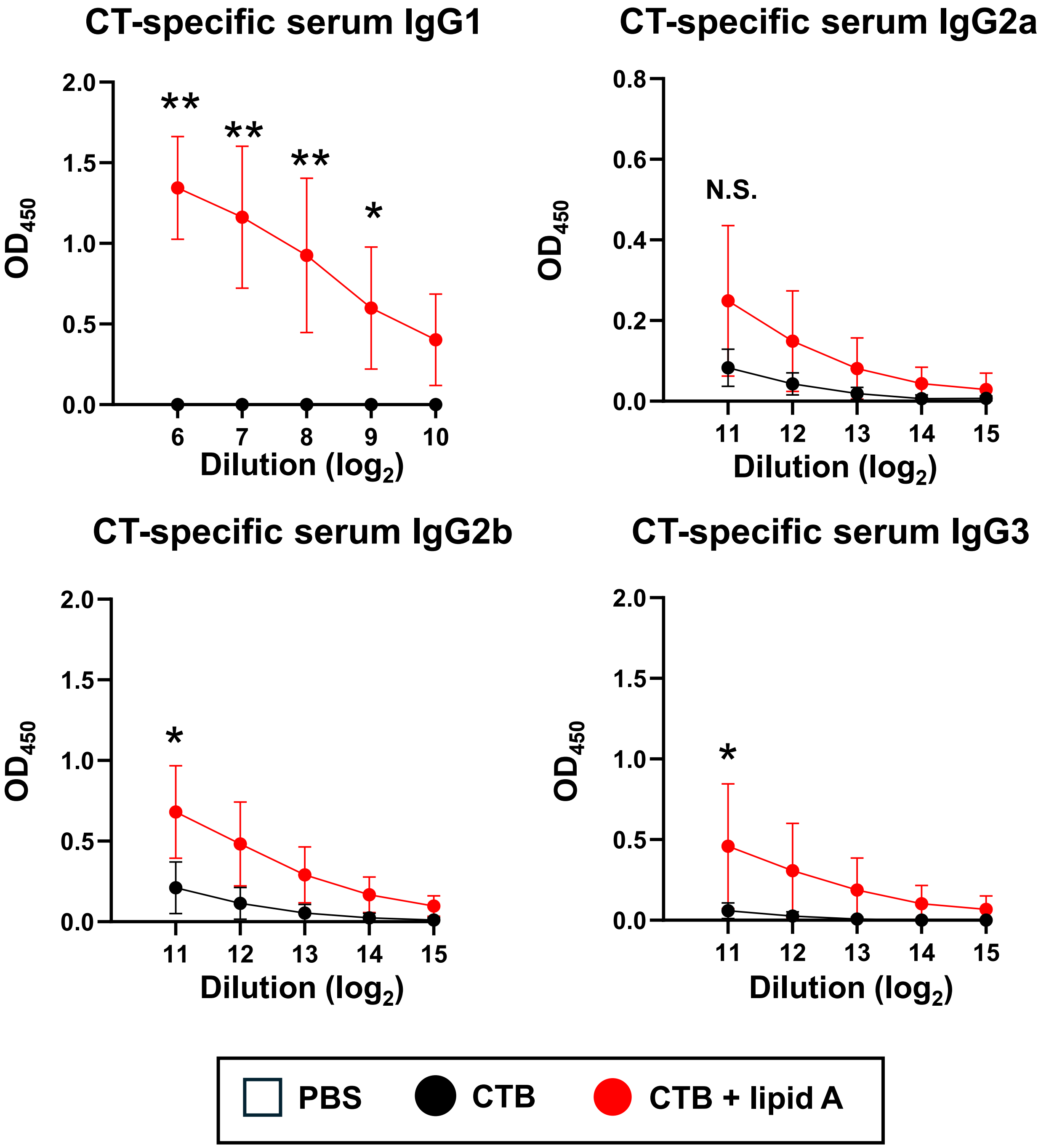

CT-specific serum IgG1
CT-specific serum IgG2a
CT-specific serum IgG2b
CT-specific serum IgG3
OD450
Dilution (log2)
**
**
**
*
N.S.
*
*
PBS
CTB
CTB + lipid A

Fig. S5

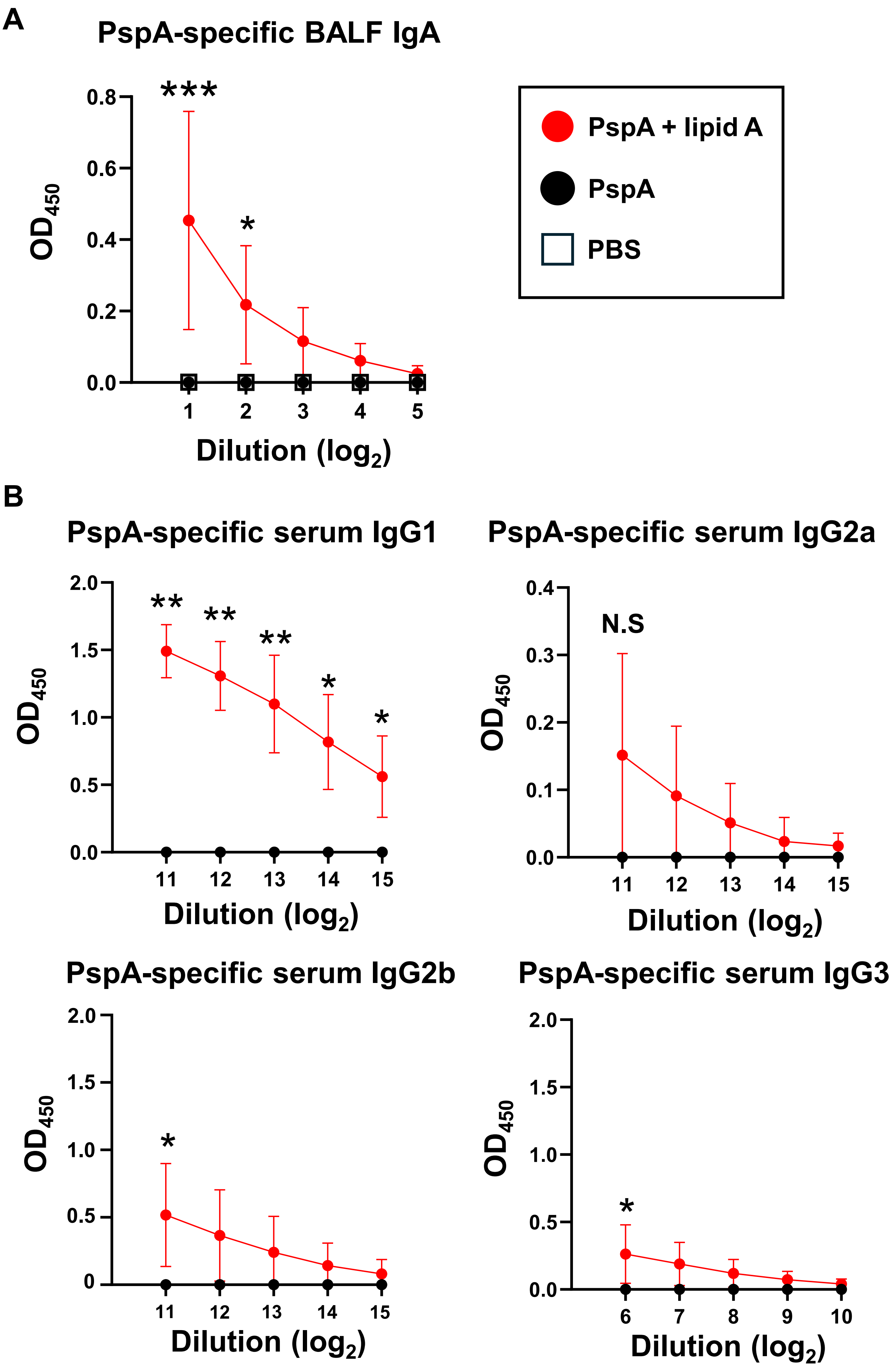
A
PspA-specific BALF IgA
***
*
OD450
Dilution (log2)
PspA + lipid A
PspA
PBS
B
PspA-specific serum IgG1
** ** ** * *
PspA-specific serum IgG2a
N.S
PspA-specific serum IgG2b
*
PspA-specific serum IgG3
*

Fig. S6

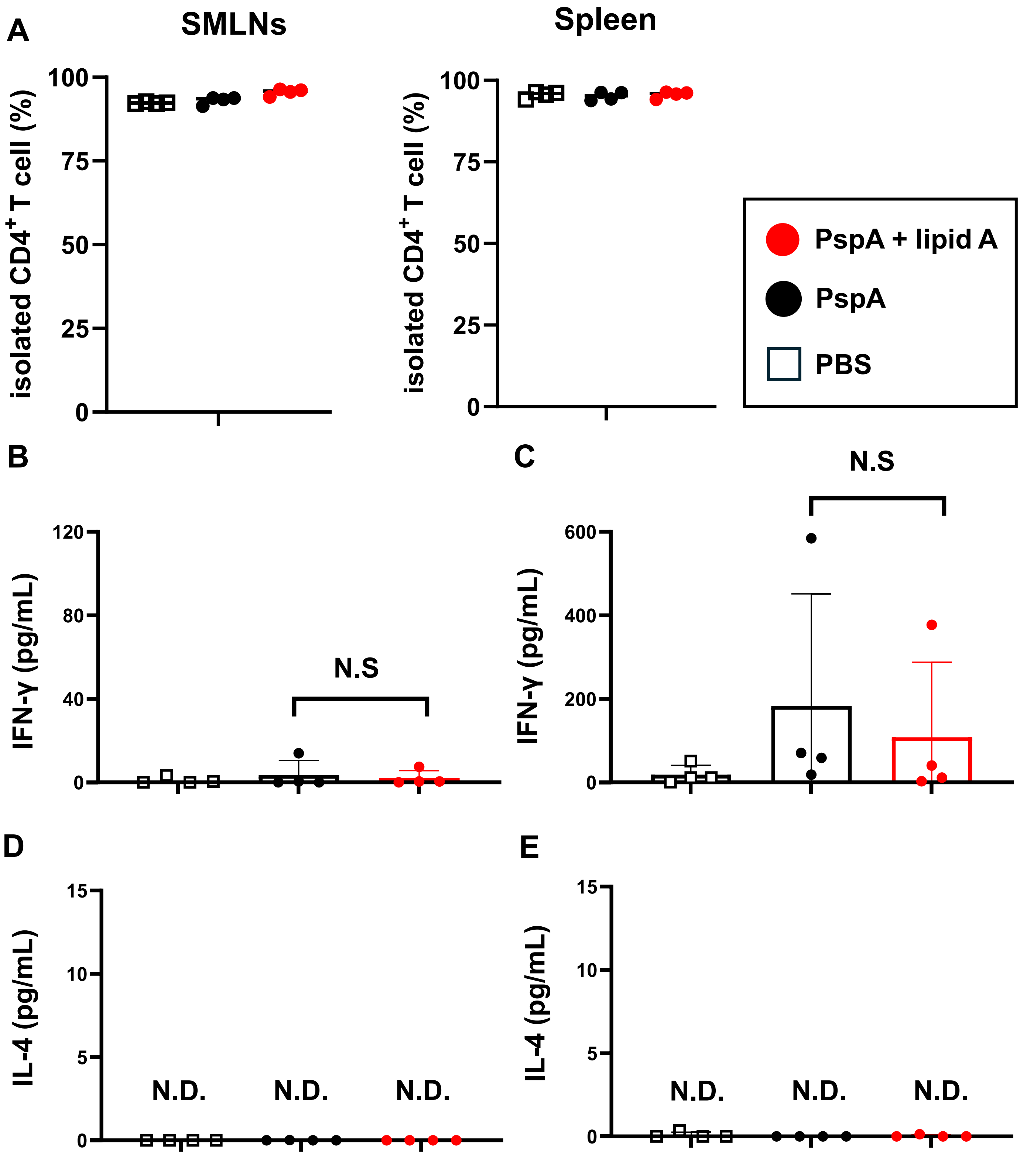
A
SMLNs
Spleen
isolated CD4+ T cell (%)
100
75
50
25
0
PspA + lipid A
PspA
PBS
B
C
N.S
IFN-γ (pg/mL)
120
80
40
600
400
200
D
E
IL-4 (pg/mL)
15
10
5
N.D.

Fig. S7

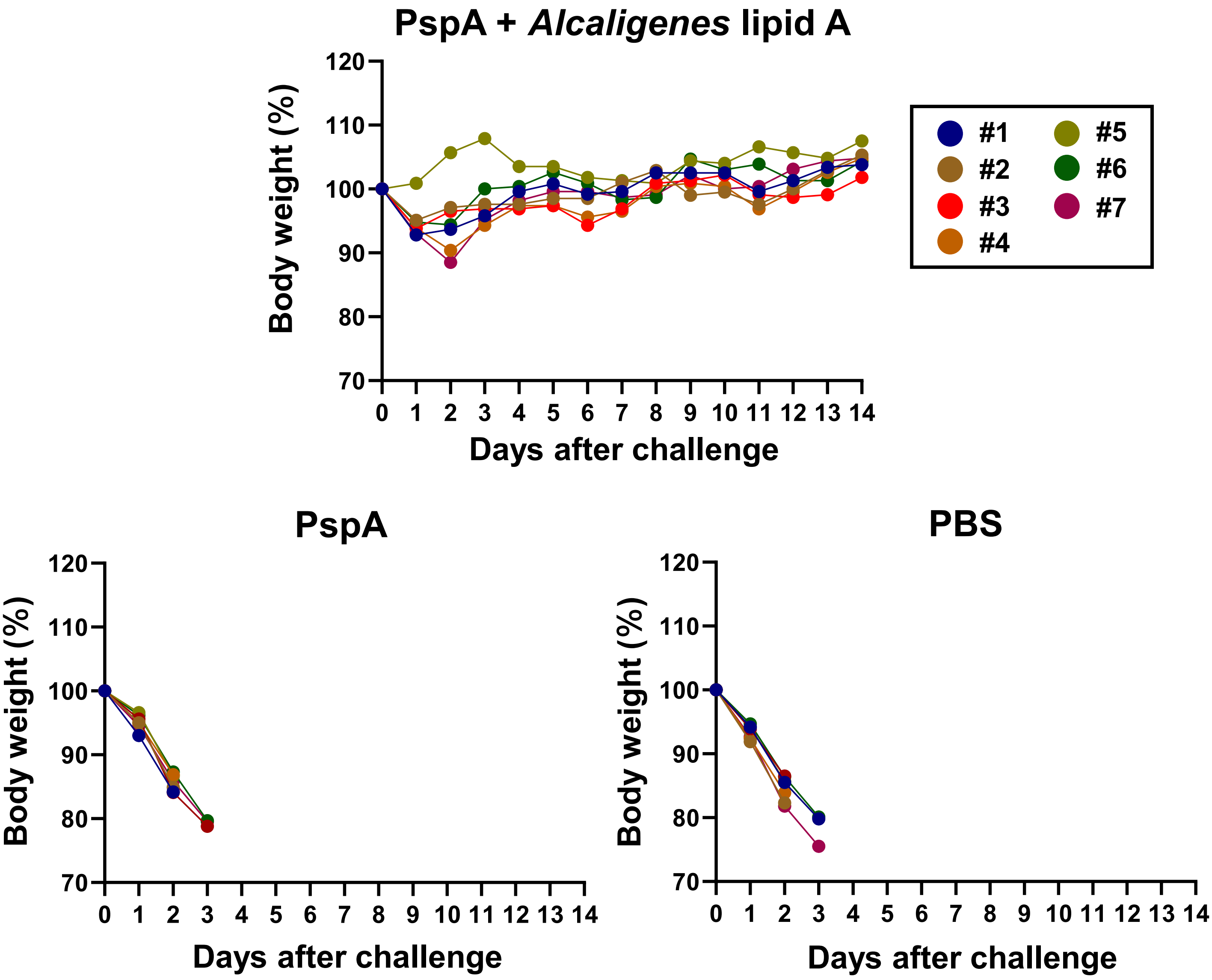
PspA + Alcaligenes lipid A
Body weight (%)
120
110
100
90
80
70
0 1 2 3 4 5 6 7 8 9 10 11 12 13 14
Days after challenge
#1
#2
#3
#4
#5
#6
#7
PspA
PBS

Fig. S8
